# Non-Invasive and Surgical Modalities for Scar Management: A Clinical Algorithm

**DOI:** 10.3390/jpm11121259

**Published:** 2021-11-29

**Authors:** Khaled Dastagir, Doha Obed, Florian Bucher, Thurid Hofmann, Katharina I. Koyro, Peter M. Vogt

**Affiliations:** Department of Plastic, Aesthetic, Hand and Reconstructive Surgery, Medical School Hannover, 30625 Hannover, Germany; Obed.doha@mh-hannover.de (D.O.); Bucher.florian@mh-hannover.de (F.B.); Hofmann.Thurid@mh-hannover.de (T.H.); Koyro.Katharina@mh-hannover.de (K.I.K.); Vogt.Peter@mh-hannover.de (P.M.V.)

**Keywords:** scar, scar therapy, algorithm, free flap, tissue transfer

## Abstract

Scars can lead to aesthetic and functional impairments. The treatment of scars requires meticulous planning and an individually adapted therapeutic strategy. A conceptual algorithm for scar treatment makes everyday clinical work easier for the practitioner and offers more safety for the patient. Based on a retrospective analysis of 1427 patients who presented for treatment of a variety of scars, we developed an algorithm for scar management and treatment. The treatments are presented using case descriptions. Additionally, an electronic search of MEDLINE, EMBASE, and ClinicalTrials.gov databases was performed utilizing combinations of relevant medical subject headings for “scar treatment”, “hypertrophic scar treatment” and “keloid treatment”. Reference lists of relevant articles and reviews were hand-searched for additional reports. Observed outcomes included: conservative scar therapy, minimally invasive scar therapy, and surgical scar therapy using local, regional and free flaps. With this work, we provide an algorithm for safe scar treatment. For better understanding, we have described a clinical case for each algorithm modality.

## 1. Introduction

Despite preventative efforts made after trauma or surgical procedures, dermal wounds will mostly heal by scarring with potentially devastating consequences e.g., emotional distress, deformity, and impaired quality of life. Patients suffering from scar tissue frequently experience stigmatization and social distress, which marks the importance of the search for efficient treatment in order to ameliorate scar tissue towards vital skin. An extensive number of techniques and treatment options exist for the management of scars. Nonetheless, the increasing prevalence of scar tissue formation and its functional and aesthetic impact remains a financial burden for the health care system. The global scar treatment market magnitude is estimated to value approximately $32 billion by 2027 [1], highlighting that scar management in the health care system will remain a significant challenge.

The concept of scar treatment ranges from simple conservative treatment options to highly complex operations that must be planned individually for each patient. The most important aim for scar treatment is the improvement of functional and aesthetical deformities [2,3]. Additionally, in order to achieve aesthetically pleasing results, homogeneous skin texture and pigmentation are essential, as well as soft tissue pliability for adequate functionality e.g., of adjacent joints [4]. Despite the efforts of clinicians and researchers, identifying successful scar treatments has remained elusive. 

Currently, there is no evidence-based gold-standard treatment algorithm for the treatment of functionally and aesthetically disruptive scars. This unsatisfactory knowledge of scar treatment is based on the complex pathophysiology, lack of suitable model systems for the evaluation of therapeutic outcomes, difficulties in quantifying changes in scar appearance, and the limited prospective, randomized controlled clinical trials of scar treatment options. Thus, patient management has been driven by clinical experience rather than adherence to a professional consensus. The last recommended algorithm was described by Gold et al. in 2014 [5]. Mostly, conservative treatment algorithms were described here without the incorporation of complex surgical scar treatment options. 

We hereby present an algorithm for scar management in the clinical setting. Each step of the algorithm is described using representative cases with simple to complex scar therapy strategies. In this article, the concepts, the algorithm for scar management, and an original catalog of indications that apply to treatments from conservative to complex operative therapies are provided based on the German and international scar management guidelines [6,7].

## 2. Patients and Methods

Based on retrospective data analysis of 1427 patients we developed an algorithm for scar management and treatment. Furthermore, an electronic search of MEDLINE, EMBASE, and ClinicalTrials.gov databases was performed utilizing combinations of relevant medical subject headings for “scar treatment”, “hypertrophic scar treatment” and “keloid treatment”. Reference lists of relevant articles and reviews were hand-searched for additional reports. Observed outcomes included: conservative scar therapy, minimally invasive scar therapy, surgical scar therapy using local flaps, and surgical scar therapy using regional- and free flaps. The treatments are presented using case descriptions.

## 3. Results

### 3.1. Conservative Treatment Options

Conservative treatment procedures such as scar massage and compression therapy using compression dressings or scar plasters containing silicone are gold standard therapies to obtain flat, soft, and aesthetically acceptable scars [8]. Furthermore, for functional rehabilitation, physiotherapy and occupational therapy remain integral treatment pillars [4]. Naturally, scar tissue shows spontaneous improvement whilst maturing. Therefore, scar revision usually will be performed after 6 to 12 months upon formation. However, conservative treatment modalities frequently will not yield satisfactory results and are often limited with regard to sustainability in complex and extensive cases, leaving surgical treatment alternatives necessary. Whether and when to perform surgical procedures is the patient’s and doctor’s joint decision upon extensive medical education about treatment alternatives and risks. The selection of an adequate surgical procedure itself remains a highly individualized concept since it is in fact dependent on a variety of factors such as the size of the scar, the texture of the skin, the anatomical region, and its pigmentation. Prior to surgery, it remains crucial to evaluate the resolution of acute tissue inflammation.

### 3.2. Laser Therapy

Laser therapy depicts a safe and effective first-line therapy in the management of traumatic scars and contractures. Early laser treatment may aid in minimizing pathological scar formation and associated disability [9]. In the treatment of hypertrophic scars, ablative lasers (10,600-nm), CO_2_ lasers, and the 2940-Er:YAG lasers, are frequently used [10]. The main goal of this treatment option is to destroy collagen and promote new collagen growth. However, treatment with ablative lasers can lead to prolonged downtime, edema, persistent erythema, keloid formation, and pigment disorders [11]. Non-ablative lasers, which also cause collagen remodeling, have fewer side effects compared to ablative lasers. Due to the superior degree of extracellular remodeling, ablative lasers generally achieve superior outcomes than non-ablative lasers. The introduction of fractional CO_2_ lasers has facilitated reduced ablative resurfacing that allows for corrections of scar surface irregularities and pliability, as well as dermal collagen reorientation in all skin types. Unlike non-ablative lasers, these CO_2_ lasers rely on a filtering system that produces microfractioned laser beams, that allow for local tissue damage with resulting ablation of the epidermis. The damaged tissue areas show spontaneous healing within 48 hours and subsequent tissue remodelling leads to flattening of scars and surface irregularities and collagen reorganization [12].

### 3.3. Triamcinolone Injections

Hypertrophic scars and keloids depict a special challenge in scar treatment. Triamcinolone acetate injections have been demonstrated to be effective short-term. Their intralesional application remains one of the most widely used treatments. Causing a reduction of collagen synthesis through fibroblast hypoactivity and a reduction in fibroblast density presumably derives their efficacy. Apart from this, triamcinolone seems to cause a decrease in endothelial bud formation from blood vessels [13]. Still, recent studies have shown, that up to 50% of keloids show no response to triamcinolone injections and may show significant relapse upon initial response [14,15]. Besides, an array of side-effects e.g., telangiectasia and tissue atrophy have been described which should not be underestimated [16]. Alternatively, the incorporation of verapamil and 5-Fluoruracil may produce superior results for medium- and long-term treatments with a reduction of given side-effects [17]. Recent clinical studies have shown that in comparison with intralesional triamcinolone treatment, combination treatment with triamcinolone and 5-fluorouracil was more effective in keloid and hypertrophic scar treatment and allowed for more significant improvement in erythema, scar height, observer assessment, and patient self-assessment. Additionally, the combination therapy offered easy administration, greater patient safety, and a reduced rate of recurrence [18]. Further studies have assessed the efficacy of oral medication on scar tissue treatment. A recent experimental study investigating the effectiveness of enalapril, candesartan, and intralesional steroid therapies in rabbits has shown that all treatment modalities were effective in the reduction of scar tissue development, whilst the best macroscopic results were obtained by triamcinolone treatment and the best microscopic results were obtained by enalapril and triamcinolone [19].

#### Case Report

**Figure 1:**
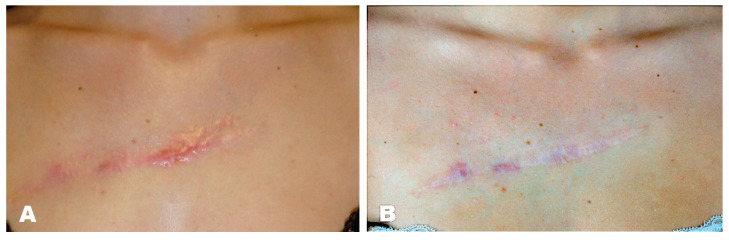
(**A**) Preoperative hypertrophic scar, (**B**) 3 months postoperative outcome after triamcinolone treatment.

A 25-year-old female patient presented with a hypertrophic scar in the area of the décolleté, which had occurred after a scratch injury sustained during her childhood. External keloid excision was performed at the age of 15 years, whereupon a scar keloid formed again. We performed triamcinolone injections in three sessions and applied silicone patches, after which the scar texture improved significantly (Figure 1).

### 3.4. Lipofilling

Autologous fat grafting as a treatment for scars includes lipofilling procedures. These are particularly indicated for patients with painful, hypertrophic, or retracted scars. The technique is usually performed under general anesthesia and is based on autologous fat extraction (i.e., abdominal fat). Upon fat processing, the injection of the remaining adipose tissue occurs in the hypodermis under the scar. In general, lipofilling procedures can be regarded as minimally invasive procedures that can contribute to a reduction of tension in scar tissue. Besides, the cosmetic appearance of a scar can be improved [20]. A disadvantage of the free fat transfer is the physiological reabsorption ranging from 10–70% of the initially injected fat grafting. Therefore, multiple sessions of fat transfer might be necessary, and permanently stable outcomes cannot be guaranteed. The technique cannot be performed in malnourished patients as fatty areas are needed for liposuction [21].

#### Case Report

**Figure 2:**
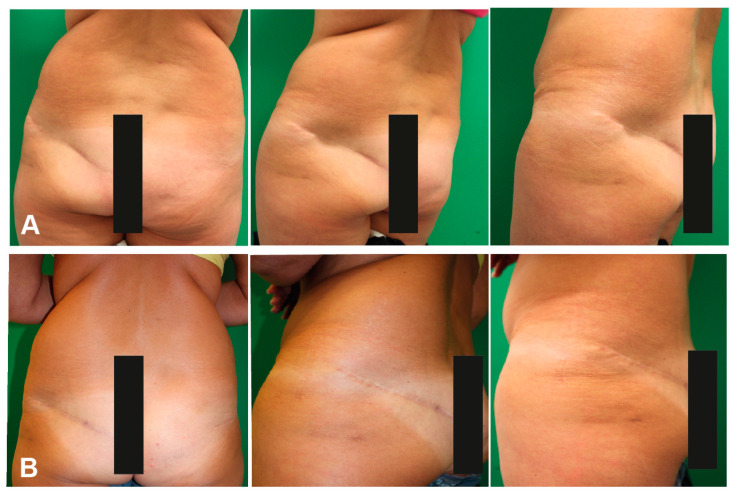
(**A**) Preoperative donor site scar after S-GAP flap harvest for breast reconstruction, (**B**) 3 month postoperative outcome after lipofilling.

This 46-year-old patient had breast ablation on the left side after breast cancer. After an unsuccessful attempt to reconstruct the breast using a deep inferior epigastric perforator (DIEP) flap, the patient’s left breast was reconstructed using a superior gluteal artery perforator (S-GAP) flap. The patient developed a retracted scar at the donor site which was painful and visible (Figure 2A). We proposed a lipofilling procedure and explained that multiple lipofilling might be necessary for a satisfactory aesthetic outcome. We performed the first lipofilling procedure (application of 337 mL, thigh liposuction). The patient was satisfied after the first complected procedure (Figure 2B). Therefore, no further lipofilling procedures were required.

### 3.5. Medical Needling

Based on the principle of percutaneous collagen induction, medical needling consists of applying needle rollers with pressure on the target area, causing an array of microwounds in the dermal layer. The procedure that can be performed under local or general anesthesia is supposed to trigger a posttraumatic inflammatory cascade whilst preserving the epidermal structures and thereby allowing skin regeneration and collagen formation [22]. Particularly the increased expression of growth factors, e.g., vascular endothelial growth factor (VEGF) and tissue growth and transforming factor (TGF-ß) is a key component of the treatment’s effect. Besides, the proliferation of dermal cells, which are crucial for skin remodeling, is initiated [23]. Apart from this, by triggering the reorganization of the extracellular matrix, the thickness of the epidermis can be shaped [24]. As an effective treatment modality for rejuvenation procedures and the treatment of wrinkles, it has advanced to a reliable method for larger scar tissue areas, which unlike laser and topically ablative treatments does not damage the epidermis. Therefore, medical needling sessions can be performed multiple times to yield optimal results.

#### Case Report

**Figure 3:**
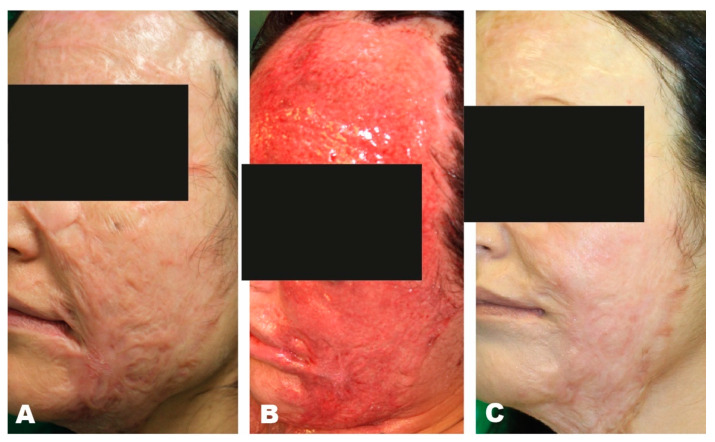
(**A**) Preoperative hypertrophic scar, (**B**) Intraoperative picture after Medical Needling, (**C**) Postoperative outcome after 3 medical needling treatments.

An 18-year-old patient suffered a car accident, sustaining 2b-3° burns to the left side of her face and décolleté. During the course of intensive medical therapy at our burn center, multiple necrectomies and split-thickness skin grafts were performed to reconstruct the burned areas. During the process, hypertrophic scarring occurred in the area of the left eye, face, and décolleté, which was associated with pain and limitation of the facial field. Initially, we performed three medical needling sessions in a row in 2017, which resulted in an improvement of the scar appearance due to collagen induction. Subsequently, in 2019, we were able to treat the visual field restriction using canthotomy and canthopexy on the left eye, as well as scar transection and full-thickness skin grafting. In the area of the décolleté, thoracic scar resection could be performed by means of expander treatment in 2019 and the reduction of the scar could be achieved (Figure 3).

### 3.6. Skin Grafting

Skin grafts provide many functional and aesthetic benefits when it comes to tissue repair. Paramount in skin grafting is the choice of split- or full-thickness grafts and choosing a sufficient texture and pigmentation match for the recipient site [25]. Whilst full-thickness grafts are particularly beneficial for covering facial defects after scar excision due to minimal skin contraction, split-thickness grafts are preferred when large defects need coverage and in areas in which skin contraction is favorable to some degree for defect size reduction [26].

The meshing of skin grafts additionally may be beneficial for instance by allowing a reduction of donor-site morbidity, by the expansion of the skin graft, and by avoiding fluid retention in infected recipient-sites [27].

### 3.7. Dermal Regeneration Templates (DRT)

Dermal templates are used when split-thickness skin grafts are not sufficient for the reconstruction of tissue defects. They provide a scaffold, which promotes tissue regeneration, immediate wound closure and depicts a physical barrier to prevent wound infections [28]. Advantages include the restoration of the skin’s pliability and mobility whilst providing sufficient sturdiness [28,29]. Types of DRTs include products derived from animal and human sources as well as scaffolds that have been artificially constructed of highly purified biomaterial or entirely synthetic polymers. For scars developing following large burn injuries, synthetic polyurethane dermal templates such as Integra (Life Sciences Corp., Princeton, NJ, USA) or NovoSorb Biodegradable Temporising Matrix (Polymedics, Denkendorf, Germany) have been established to address the lack of autologous skin to graft and to restore the dermal skin layer [30]. Integra presents a decellularized dermal template derived from animal sources and consists of purified collagen from bovine tendons crosslinked with glycosaminoglycan obtained from shark cartilage and may be supplied with a removable silicone layer that functions as a temporary epidermis [30]. In contrast, BTM is a fully synthetic dermal template which consists of biodegradable polyurethane foam with a temporary non-biodegradable polyurethane seal [31]. Compared to Integra, BTM evades the risk of cross-species immune rejection or disease transmission, as well as circumvents ethical and cultural objections to using animal-derived products [28].

BTM application has shown to be successful in improving scar quality upon application and limiting wound contraction significantly [32,33]. Its reconstructive possibilities can be used for a variable range of tissue defects that are not responsive to instant skin grafting. Key advantages are the vascularization and integration of the template even in the presence of infection, thereby allowing an application in patients with an array of comorbidities [34].

#### Case Report

**Figure 4:**
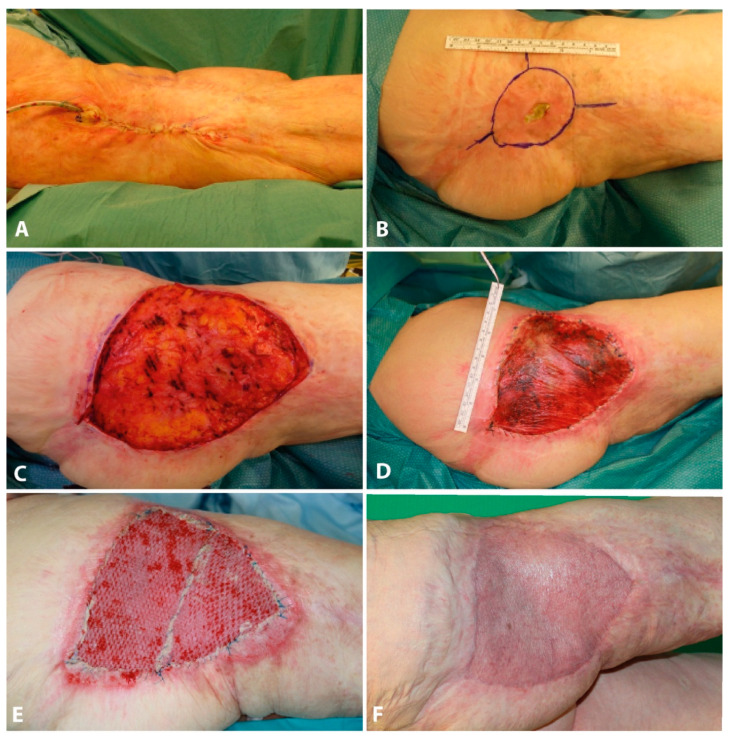
(**A**) Postoperative picture after scar excision and primary wound closure, (**B**) Postoperative development of a wound healing disorder, (**C**) Another scar excision, (**D**) Application of BTM, (**E**) Split-skin graft 3 weeks after BTM application, (**F**) Final outcome.

A 79-year-old woman with a deep burn injury scar on the right lateral thigh was referred to our center. She had suffered from a scalding injury in her early childhood. The initial injury was treated conservatively. In 2017, the patient underwent surgical excision of keloids in the formerly burned area. Subsequently, she developed a wound-healing deficit and wound dehiscence. The following treatment comprised of wound debridement, scar excision, and primary wound closure shortly after. Three-months postoperatively, the patient had presented with a recurrent wound dehiscence that was surgically revised by wound debridement and skin advancement flap. After 14 days, the wound had healed sufficiently. The representation of the patient occurred three years later with a flap skin dehiscence. Sufficient healing could not be achieved by conservative treatment with disinfecting topicals. Additionally, the patient described an uncomfortable and painful feeling of tension in the formerly burned areas. Physical examination showed a predominantly non-irritated extensive scar area of approximately 20 × 10 cm on the lateral aspect of the right thigh with scar tissue extending into the gluteal and trochanter region. On the latero-proximal border of the scar was an approximately 3 × 1 cm sized wound dehiscence. We performed another wound debridement and opted for extensive scar excision and application of Novosorb BTM. Efforts were made in order to ensure sufficiently vascularized subcutaneous tissue. The wound was sealed by vacuum-assisted closure (VAC) dressing. On days 8 and 16 the patient was taken back to theater for BTM evaluation and VAC dressing change. On day 21 we performed the surgical delamination of the BTM. It showed full adhesion to the underlying wound bed. We went on to perform split-thickness grafting over the delaminated BTM. At 1-month follow-up, the reconstructed area presented with a natural appearance with flexible skin and minimal skin contraction. The uncomfortable feeling of tension was fully regressive, and the patient’s mobility was fully preserved. Post-dressing removal, we initiated physiotherapeutic rehabilitation for flexibility and endurance (Figure 4).

### 3.8. Local Flaps

Local flaps, such as Z-plasties, can be performed in order to achieve reorientation of scar tissue and to position it further into relaxed skin tension lines. Surgically it is accomplished by performing a double transposition local flap, positioning the central limb of the Z-platy perpendicularly to the former scar [35]. Advantages also include the breaking up of scar tissue resulting in an irregularization, which renders scar perception less conspicuous. Presumably, the Z-plasty’s variety of tension vectors may aid in the prevention of scar contraction and hypertrophy.

For extensive scars, Z-plasties can be performed with multiple and continuous incisions. Disadvantages that go in hand with performing Z-plasties include the increase of the scar’s lengths as well as the addition of scar lines, which partially cannot be positioned within relaxed skin lines.

#### Case Report

The patient suffered a severe childhood burn affecting the trunk, thighs, and right upper arm including the axilla (30% total body surface). The initial therapy consisted of necrosectomy with skin grafting. Further, three sessions of medical needling were performed to improve the scar pattern. The patient presented to our special consultation hour complaining about a hypertrophic scar with local contracture in the right axilla.

During clinical examination the patient was unable to abduct his right shoulder >90° due to contracture of a hypertrophic scar measuring approximately 30 cm. Operative resection of hypertrophic scar tissue was performed under general anesthesia. Wound closure was performed using Z-plasties in order to achieve a free range of motion of the right shoulder. Postoperatively the patient received a Gilchrist sling for seven days and was discharged after four days.

**Figure 5:**
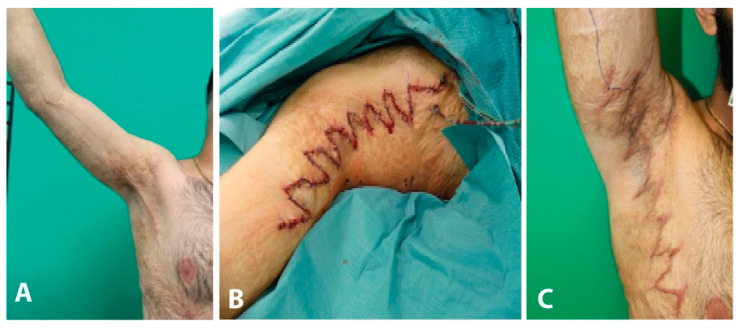
(**A**)Preoperative hypertrophic scar with functional limitation of the arm, (**B**) Intraoperative picture after Z-plasty, (**C**) Postoperative outcome.

The patient returned to our consultation hour three months postoperatively. The scar healing was smooth with no hypertrophic areas. Range of motion for the right shoulder was without any restriction in all planes (Figure 5).

### 3.9. Expander

Advantages of gaining skin and soft tissue using expanders include suitable aesthetic results due to the superior skin quality and color match and no donor-site morbidity.

They are usually used for reconstruction in the area of the scalp, face, chest, and extremities. The soft tissue obtained by expanders can also be used as a pedicled flap to cover defects of the head, neck, or face area. For this purpose, pre-expanded supraclavicular flaps or super thin posterior thorax flaps can be used, too [36]. However, the practitioner should be aware that the expander can severely limit the functioning of the patient, cause pain, and cannot be tolerated by all patients. In case that all above-mentioned options are not applicable, free flaps are indicated to cover the tissue defect after scar excision [37].

#### Case Report

The patient suffered a scalding injury by boiling water affecting the chest with contracture of the upper pole of both breasts 20 years ago. Previously, multiple sessions of medical needling were performed in our clinic. However, the patient complained about a persisting hypertrophic scar contracture. An area of hypertrophic scar tissue measuring 10 × 12 cm was identified. Due to scar contracture, the upper breast poles were drawn upwards.

In order to achieve distension of fibrous scar tissue, two subcutaneous expanders (55 mL each) were placed presternally using a horizontal incision. The expanders were explanted two months postoperatively. During the same surgery, the hypertrophic scar area was resected completely, and wound closure was achieved using a V-shaped advancement flap.

**Figure 6:**
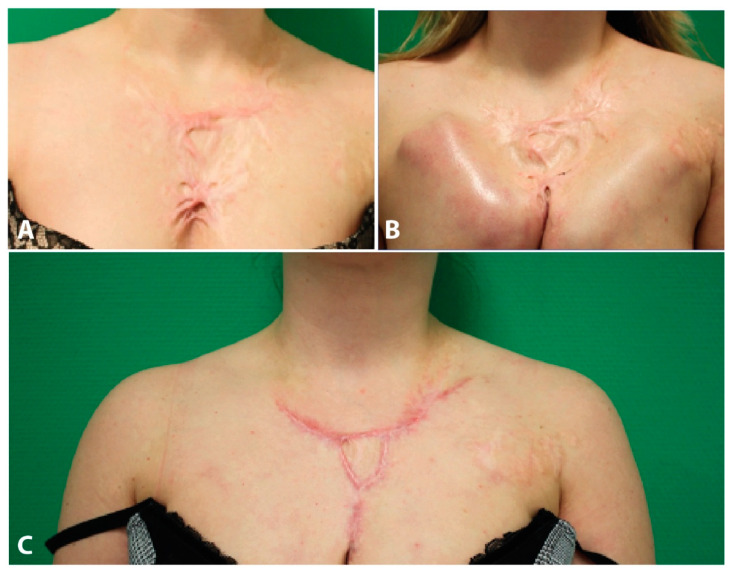
(**A**) Hypertrophic scar in the chest area, (**B**) After expander implantation, (**C**) Outcome after 6 month.

The patient was satisfied with the functional and aesthetic result. Further on, a local lipofilling was performed to improve the local scar condition (Figure 6).

### 3.10. Vascularized Flaps

Regional flaps such as vascularized pedicled fasciocutaneous or myocutaneous flaps are applied if there is a lack of adjacent tissue to cover the defect after scar excision [38,39]. Due to the complexity of these operations and the risk of donor-site morbidity, regional flaps are only indicated in cases of severe functional or aesthetic impairment. The most commonly used pedicled flaps include groin flaps for covering defects in the hand area or transverse rectus abdominis myocutaneous (TRAM) flaps for covering tissue defects in the lumbar and hip areas. In the case of the inguinal flap, it should be noted that a two-stage surgical treatment is necessary. Multistage treatment strategies e.g., serial scar excisions tissue expander are indicated if the expanded soft tissue has better quality compared to other options and the patient does not mind undergoing multiple interventions [36].

#### Case Report

**Figure 7:**
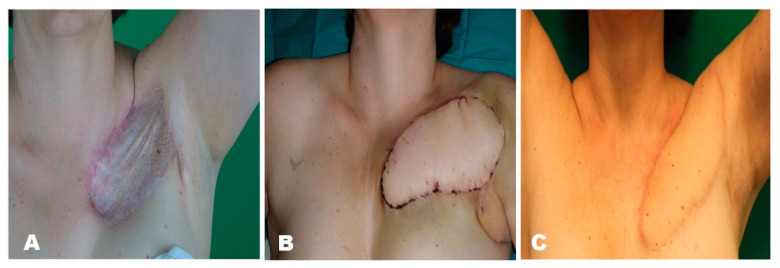
(**A**) Scar after skin graft transplantation, (**B**) 7 days postoperatively after pedicled latissimus dorsi muscle flap transfer, (**C**) Postoperative outcome after one year.

The patient had breast cancer (right side) in 2011. She got neoadjuvant chemotherapy (Epirubicin) via a port catheter. During the first cycle of chemotherapy, the patient suffered from an extravasate. However, no surgical therapy was needed, and the tissue was healing. After completion of all chemotherapy cycles, the patient got a breast-conserving surgical treatment, an axillary lymph node dissection (level 1 and 2) and radiation. Subsequently, the port got removed and the patient suffered from a wound-healing disorder. Multiple surgical wound debridements were performed including VAC-therapy and the application of a split-skin graft. However, the patient developed a massive and painful scar contracture in the chest area. The scar was excised and a musculus latissimus dorsi pedicle flap was performed. As the pedicel flap was prominent over the skin level, the patient received liposuction of the pedicle flap. The patient further got physiotherapy and conservative scar treatment. Because of a relieving posture, the patient developed a shoulder contracture in 2018 and needed physiotherapy on a regular basis. (Figure 7).

### 3.11. Free Tissue Transfer

Due to technical advances in medicine and the improvement of microsurgical operating techniques, free tissue transfer has emerged as one of the most effective therapy methods for large wounds. Because of the better understanding of the free flap plasty, e.g., flap geometry, blood supply, and flap delay, new options were created to provide a wide range of tissue that could be transferred to the most distant locations of the human body. All types of tissues, including bone, tendon, muscle, fascia, fat, and skin can be used as free tissue transfer (Table 1). The pattern of the vascular supply determines the size, design, and thus the individual requirements for covering tissue defects. If there is no recipient vessel available e.g., in case of vascular diseases, severe scarring or irradiation, venous grafts or arteriovenous loops can be applied to provide recipient vessels for free flaps [36]. Due to the anatomical variations, the flap architecture and its vascular pattern are variable. Therefore, an initial magnetic resonance imaging (MRI) or computed tomography (CT)-angiography could help to develop an adequate strategy for applying free tissue transfer. Profound anatomical knowledge about the muscle origin, its insertion as well as vascular supply like perforator location or location of recipient’s vessels is essential to provide the right indications for a free flap. We provide a systematic approach of free flaps regarding tissue requirements after scar excision (Table 1).

#### 3.11.1. Case Report 1

**Figure 8:**
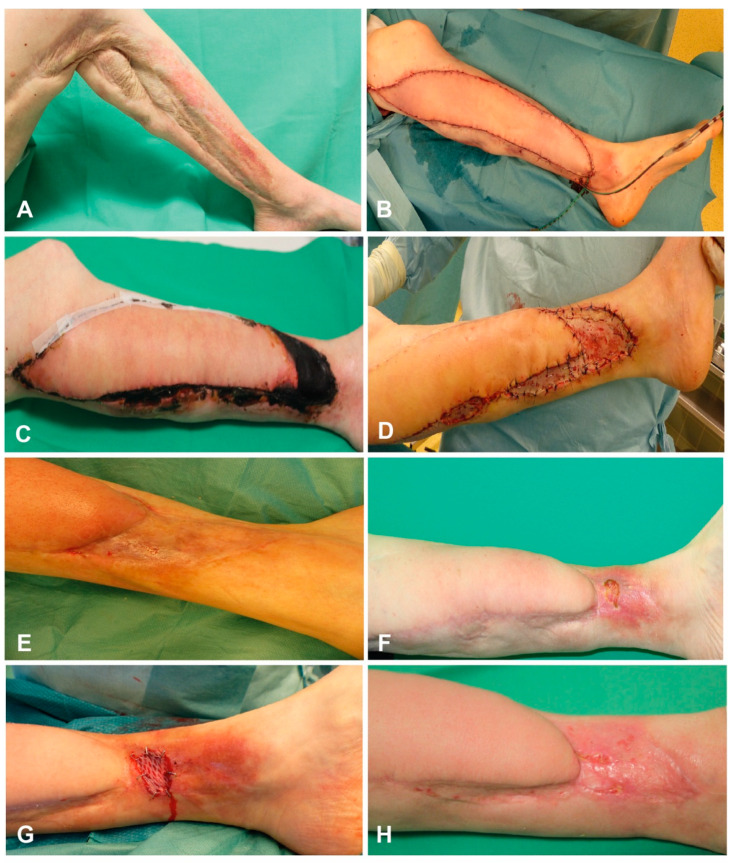
(**A**) Fragile and painful scar of the left leg, (**B**) Intraoperative picture after excision of the scar and reconstruction of the lower leg using latissimus dorsi muscle free flap, (**C**) Development of flap margin necrosis, (**D**) Excision of the flap margin necrosis and skin graft transplantation, (**E**) complete healing of the wound, (**F**) Development of a small wound, (**G**) Treatment of the wound using skin graft, (**H**) Final outcome.

In 2003 the patient suffered from necrotizing fasciitis. Subsequently, the patient got a split-skin graft. However, the patient developed a fragile and painful scar on the left lower leg. In May 2013 the patient received a musculus latissimus dorsi free flap (MLD) after excision of the scar. Postoperatively, a small part of the free flap got necrotic. A debridement and removal of the necrotic tissue was necessary. However, a secondary wound closure was not possible, and a full-thickness skin graft and VAC-therapy were applied in June 2013. After VAC-therapy, another full-thickness skin graft was needed. In November 2014, lipofilling between the MLD and the split skin graft was performed. In September 2018, the patient suffered from a minor trauma at the lower leg and needed another split skin graft. Afterwards, the patient was satisfied and experienced no further complications (Figure 8).

#### 3.11.2. Case Report 2

**Figure 9:**
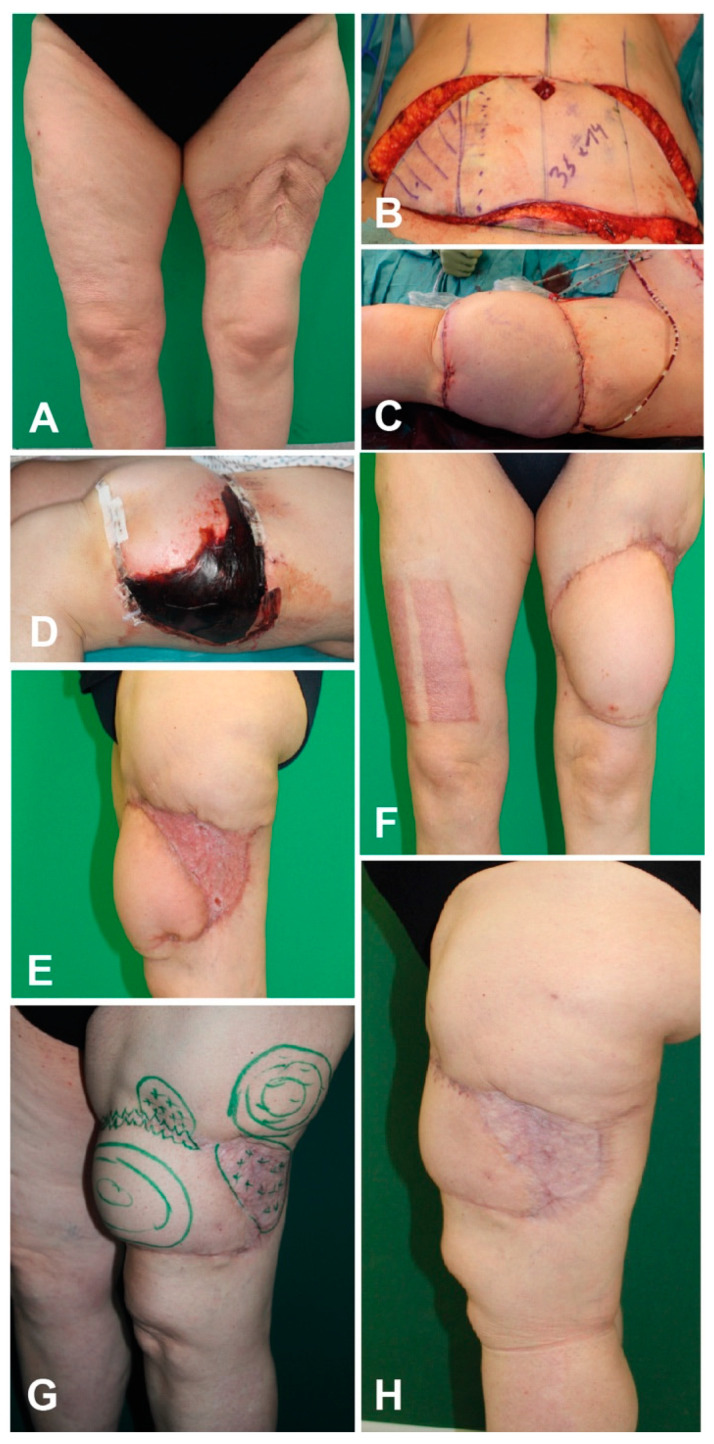
(**A**) Split-thickness skin graft scar, (**B)** Preparation of a DIEP flap, (**C**) Covering of the soft-tissue defect after scar excision, (**D**) Development of flap margin necrosis, (**E**,**F**) Postoperative image after excision of flap necrosis and skin graft transplantation, (**G**) Preoperative planning of liposuction of the flap, (**H**) Final outcome.

A 59-year-old patient suffered from multiple traumas including a femur fracture, ankle fracture type Weber B on the right side, commotio cerebri, and soft tissue defect anterior to the left thigh. The fractures were treated by trauma surgeons and the soft tissue defect was covered using a skin graft. Three years later the patient presented to our clinic with a scar in the area of the split-thickness skin graft. Since the skin graft was attached directly to the muscle, the scar was concerning functionally and aesthetically. To adequately reconstruct the soft tissue of the left thigh, the scar was excised and the wound was subsequently covered using a free DIEP flap. However, the flap developed marginal necrosis. After debridement of the necrosis, the remaining defect was covered using a split-skin graft. 1 year later the DIEP flap was thinned out, followed by a liposuction of the flap to improve contour. Finally, the flap and its adjacent area were aligned using liposuction (Figure 9).

## 4. Discussion

**Figure 10:**
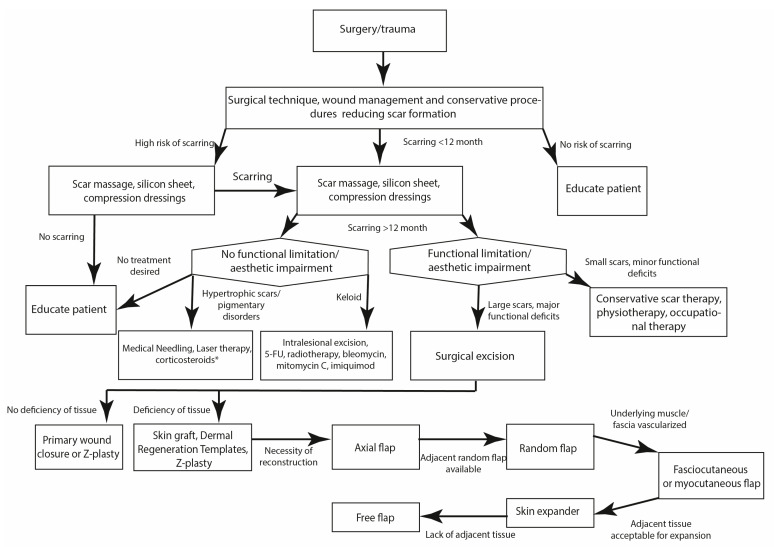
Algorithm for the treatment of scars.

The prevalence of scar tissue formation is on the rise. However, successful scar management has still remained elusive for many clinicians in light of a broad array of possible treatment modalities and the continuous evolution of new therapeutic options. Multiple well-established therapeutic modalities to treat scars have been described and treatment advances have allowed us to improve aesthetic and functional deficits due to scars. Efficient comprehensive scar treatment frequently incorporates conservative measures, physical therapy, injection of corticosteroids and antimetabolites, laser treatment, and surgical options. Based on our clinical experience with the presented scar treatment modalities, we have attempted to compose a treatment algorithm for the management of scars, in the hope that this paradigm will aid clinicians in their treatment evaluation. Based on the considerations depicted above, we present an extensively applicable algorithm for the treatment of a variety of scars (Figure 10).

During the initial consultation, we firstly aim to inform the patient of all the possible treatment options and their realistic outcomes. Early on, we highlight the frequent requirement of several treatment sessions and the possibility of remnants or recurrence of scar tissue. In general, patients presenting with scars prior to full scar maturation (12 months postoperatively/after trauma) are advised to take full advantage of available conservative treatment options comprising of scar massage, the application of silicone sheets and compression dressings. Particularly with an early application, this may aid in optimizing the aesthetic factor of scars and may render further treatment modalities unnecessary. Upon scar maturation and absence of scar regredience, scar re-evaluation takes place. In the case of remaining aesthetic impairments or hypertrophic scars, the application of laser therapy or corticosteroid injections may be a viable treatment option. For larger scar areas, such as post-burn scars or extensive self-injury scars, medical needling may be a promising treatment option, despite its invasiveness and the need for regional or general anaesthesia. Patients presenting with keloids usually undergo intralesional keloid excision and triamcinolone and antimetabolite injection. In our clinic, postoperative radiotherapy has been an integral step postoperatively to help minimize keloid recurrence.

In case of extensive functional deficits resulting from the scar tissue, we recommend scar excision and subsequent primary wound closure, when feasible. To reduce tension on the new scar, Z-plasties or skin grafts may need to be performed. Adhering to the principles of the reconstructive ladder, larger defects resulting from scar excisions will have to be addressed by local, regional or free flaps.

Naturally, patient education remains an integral part of the joint decision-making process throughout each step of the algorithm and all therapeutic measures should be based on a highly individualized approach to the patient’s impairments and wishes. In clinical practice, the therapeutic endpoint is mostly based on patient satisfaction regarding scar improvement. Evidence-based therapeutic concepts should remain an integral part of the decision-making process as well as patient-dependent parameters.

## Figures and Tables

**Table 1:** Free flaps regarding tissue requirements after scar excision.

Flap Type	Included Tissue	Examples	Blood Supply	Size
Arterial	Skin, subcutis and fascia	Radial arm flap	Radial artery	8 × 16 cm
		Dorsalis pedis flap	Dorsalis pedis artery	3 × 7 cm
Muscle or myocutaneous	Skin, subcutis, muscle	Latissimus dorsi	Thoracodorsal artery	Muscle: 20 × 40; Skin: 12 × 20
		Rectus abdominis	Superior or inferior epigastric artery	Muscle: 25 × 6 cm Skin: large traverse or vertical paddle
Fascial, adipofascial and fasciocutaneous	Skin, subcutis, fascia	Serratus fascia	Serratus branch of thoracodorsal artery	12 × 16 cm
		Parascapular flap	Descending branch of the circumflex sapular artery	15 × 25 cm
Perforator	Skin, subcutis and scubcutaneous fascia	Anterolateral thigh flap	Septocutaneous perforators from descending branch of the lateral femoral circumflex artery	8 × 25 cm
		Deep inferior epigastric perforator flap	Deep inferior epigastric artery	Variable skin paddle, similar size to TRAM
Specialized	Sensor tissue, differentiated structure and texture	Wrap around flap	First dorsal metatarsal artery	Big toe
		Composite tissue allografts	No blood supply	e.g., finger tip

## Data Availability

The data presented in this study are available on request from the corresponding author.
